# Extracts from the Report of a Committee of the New York Academy of Medicine, upon the Comparative Value of Milk Formed from the Slop of Distilleries and Other Food

**Published:** 1851-12

**Authors:** 


### ECLECTIC DEPARTMENT,

### AND SPIRIT OF THE MEDICAL PERIODICAL PRESS.

---

*Extracts from the Report of a Committee of the New York Academy of Medicine, upon the comparative value of Milk formed from the slop of Distilleries and other food.*

[From the published volume of the transactions of the New York Academy of Medicine, just issued, we extract the following, the bearings of which upon the public health need no arguments from us to enforce them. It will particularly claim the attention of city readers.—*Ed. N. Y. Jour. Med.*]

In investigating this matter, your committee have not forgotten the importance of careful attention, accurate and repeated examinations, unswayed by any bias. The importance of such an investigation of an article which constitutes so large a portion of the food of the inhabitants of this city, is evident to all. In 1842 it was estimated that the quantity of milk daily used in the city of New York amounted to more than 15,000 gallons, at that time principally furnished from the neighborhood. With the growth of the city, the consumption of milk has undoubtedly increased. The various kinds of milk which are consumed in the city, may be divided into several classes, according to its origin.

1st. The grass-fed milk, brought from a distance by steam — as the Orange county milk, for example.

2d. That produced by feeding the cows entirely, or partially, with distillery slop, which is the refuse of the grain left after distillation, mixed with water, with most or all the spirituous ingredients extracted, brought hither by steam — as, for example, the Newburg.

3d. Grass-fed milk produced in the neighborhood of the city, as some of the Bloomingdale dairy milk.

4th. That produced partially by the administration of distillery slop, in the neighborhood, as at Brooklyn, Wallabout, Bloomingdale, &c.

5th. That produced in the city, or its outskirts, solely by feeding on distillery slop, as at the distilleries on Long Island, Sixteenth and Forty-Second streets in this city. * * * * * *

Your committee have endeavored to take nothing for granted, but have striven to verify, by repeated observations, the truth or falsity of every statement, and endeavored to guard against any fraud. For this reason, every specimen of milk examined, has been personally obtained by the chairman of this committee, from the milkers themselves, and placed in the hands of the chemist. Any adulterations or additions have been made, therefore, by the producers themselves, and are not attributable to any of the retail venders. The distillery milk has been obtained from Johnson's Distillery, Sixteenth street, and Tenth Avenue. The Orange county milk was bought at the depot, at the foot of Duane street, without the knowledge of the proprietors of the object for which it was procured, and taken from the same can from which other customers were served. It, therefore, was not enriched with additional cream, for the purpose. * * * *

The chemical examinations were entrusted to Mr. Lawrence Reid, whose skill as an analytical chemist is well known to the members of the Academy. He has devoted much time and attention to the investigation, proposing and using new tests of its virtues, some of which will be hereafter mentioned, and displaying that zeal in the cause of science, which strongly recommends him to the favorable consideration and thanks of the Academy.

For the microscopical investigations, the Academy is indebted to the practiced eye and sound judgment of Dr. Alonzo Clark, Professor of Pathology in the College of Physicians and Surgeons, in this city. For his readiness in so materially assisting in this investigation, Dr. Clark will undoubtedly receive the acknowledgments of the Academy, as he has already the personal thanks of the committee.

The reports of these gentlemen now follow in full.

New York Hospital, *Nov. 29th*, 1847.

Sir:—At the request of the committee of the Academy of Medicine, on milk, I have made several analyses of distillery and other milks, and have the honor to report:

1st. That of the various samples furnished me, none of the common sophistications were present, such as starch or carbonate of lime, and that, as far as chemical tests could enable me to determine, the samples were all genuine.

2d. That with the exception of the sample marked No. 6, of the analyses, they were all of a neutral character, when received, No. 6 being acid.

3d. With regard to the assertion that distillery milk contains spirits, the milk has been tested on such a scale as to detect one fifty-thousandth part of spirits, if present, and none could be found.

4th. With regard to distillery milk furnishing no butter, it is a mistake, as every sample, sixteen, gave butter, on agitation in a bottle. The butter is whiter, and in much smaller quantity than obtained from other milk, and this may be considered a good test of distillery milk, the scantiness of the butter. The butter, also, in forming, associates itself with more curd and whey than that obtained from other milk. To examine milk, for butter, a bottle containing a pint may be filled two-thirds with milk, and well shaken for about an hour and a half, when the butter will separate.

5th. With regard to the relative proportions in which the ingredients exist. The nature of fermentation and distillation is, to abstract from the grain all the fecula and sugar, the principles that are more particularly convertible into butter and sugar; leaving the nitrogenized compounds, and also the caseine and earthy matter nearly untouched; hence the increased quantity of ashes and also of caseine, the nitrogenized compound in milk; while the sugar and butter are below the usual standard.

6th. To test if this milk was decomposed by heat in the same manner and time as other milk, a portion was placed in a glass vessel, and retained at the temperature of 98 degrees of heat, for six hours before coagulation took place, while a portion of Orange county milk, treated in the same manner, coagulated in one hour.

Whether this experiment is to be considered as having a bearing on the assimilation of the milk as food in the human stomach, I prefer leaving to the committee to decide.

The samples are marked from one to six. No. 1 is a European analysis of milk, by M. Haidlen, the most recent I could find. No. 2 is Orange county milk, being an average of two samples. Nos. 3, 4, 5, 6, are distillery milk, about sixteen samples of which were examined.

Yours respectfully,
LAWRENCE REID,
*Prof. of Chemistry to College of Pharmacy.*

A. K. GARDNER, M. D.,
*Chairman of Com. of the Acad. of Med. on Nuisances.*

ANALYSES OF MILK.

| | No. 1. | No. 2. | No. 3. | No. 4. | No. 5. | No. 6. |
|---|---|---|---|---|---|---|
| Water, | 873.00 | 860.00 | 869.10 | 876.00 | 888.00 | 898.00 |
| Butter, | 30.00 | 35.00 | 15.00 | 14.00 | 13.00 | 10.00 |
| Caseine, | 48.20 | 45.00 | 62.00 | 59.00 | 50.00 | 45.00 |
| Sugar of Milk, | 43.90 | 53.00 | 44.00 | 42.00 | 41.00 | 40.00 |
| Phosphate of Lime, | 2.31 | 3.35 | 4.20 | 4.00 | 3.20 | 2.80 |
| " Magnesia, | .42 | .76 | 1.84 | 1.56 | 1.41 | 1.20 |
| " Iron, | .07 | .09 | .12 | .11 | .10 | .07 |
| Chloride of Potassium, | 1.44 | 2.00 | 2.97 | 2.51 | 2.46 | 2.23 |
| " Sodium, | .24 | .36 | .44 | .42 | .43 | .40 |
| Soda in Combination with the Caseine, | .42 | .50 | .43 | .40 | .40 | .30 |
| | 1000. | 1000. | 1000. | 1000. | 1000. | 1000. |

MICROSCOPIC EXAMINATION.

DEAR DOCTOR:— I have examined, with the aid of the microscope, the four specimens of milk you sent me. Four statements will comprise all that I observe in them worthy of remark:

1st. The milk-globules are less abundant in them than in specimens of good country milk, with which they have been compared.

2d. The globules, though very variable in size, are generally smaller than in good milk.

3d. In three of the specimens there was an unusual tendency to aggregation in the globules, and when once aggregated, they adhered in groups, so that mere agitation would not separate them.

4th. In the last two specimens there was an unusual number of epithelial cells,* many of which were very markedly granular, and some highly colored — in a few of these the milk-globules were still imprisoned, and of very

* "The cells covering the simple membranes that form the free surfaces of the body, whether external or internal, are all entitled to be regarded as *secreting* cells; since they separate from the blood various products which are not again to be returned to it." (Carpenter's "Manual of Physiology," p. 408.)

"From the researches of Mr. Goodsir, it appears that, in common with other glandular structures, the inner surface of the milk-follicles is covered with epithelial cells, which, being seen to contain milk-globules, may be, without doubt, regarded as the real agents in the secreting process." (Carpenter's Physiology, Clymer's ed. 1847, p. 649 — see also "Anat. and Path. Obs.," by John and Harry D. S. Goodsir, p. 24.)

small size; showing that these secreting structures had been discharged from the lactiferous ducts of which they form the lining, before the complete elaboration of their contents.

Your familiarity with the subject to which these statements relate, will render any comment on my part unnecessary. I submit them, therefore, as the basis of any remark which your wider range of research may justify.

Your ob't servant,

*New York*, *Nov.* 27, 1847. A. CLARK.

To Dr. Gardner.

